# The self-regulated learning of medical students in the clinical environment – a scoping review

**DOI:** 10.1186/s12909-017-0956-6

**Published:** 2017-07-10

**Authors:** Kenneth K. Cho, Brahm Marjadi, Vicki Langendyk, Wendy Hu

**Affiliations:** School of Medicine, Western Sydney University, Campbelltown, NSW 2560 Australia

**Keywords:** Self-regulated learning, Clinical years, Clerkship, Scoping review

## Abstract

**Background:**

Self-regulated learning is the individual’s ability to effectively use various strategies to reach their learning goals. We conducted this scoping review to explore what has been found regarding self-regulated learning in the clinical environment and how this was measured.

**Methods:**

Using Arksey and O’Malley’s five-stage framework, we searched three medical and educational databases as well as Google Scholar for literature on the self-regulated learning of medical students in the clinical environment published between 1966 and February 2017. After results were screened and relevant studies were identified, the data was summarised and discursively reported.

**Results:**

The search resulted in 911 articles, with 14 articles included in the scoping review after the inclusion criteria was applied. Self-regulated learning was explored in these studies in various ways including qualitative, quantitative and mixed methods. Three major findings were found: 1) levels of self-regulated learning change in the clinical environment, 2) self-regulated learning is associated with academic achievement, success in clinical skills and mental health and 3) various factors can support self-regulated learning levels in medical students.

**Conclusions:**

Most of articles exploring the self-regulated learning of medical students during the clinical years have been published in the last 5 years, suggesting a growing interest in the area. Future research could explore the self-regulated learning levels of medical students during the clinical years using a longitudinal approach or through the use of novel qualitative approaches.

## Background

Self-regulated learning (SRL) is the process where one is ‘metacognitively, motivationally, and behaviourally proactive in the learning process’ [1]. More specifically, self-regulated learners: (i) monitor their own progress towards self-set goals and are therefore able to reflect on the effectiveness of their learning approaches; (ii) tend to view the learning task as intrinsically interesting and worth-while, and have high levels of self-efficacy, and (iii) engage in and persist with learning behaviours that maximise the degree to which learning occurs [2]. The concept of SRL has been found to be relevant to high-school and university students, educators and policy makers [3, 4]. In medical education, with the need for physicians to be life-long learners, there has been a push for the development of SRL [5].

According to some theories, SRL can be categorized into four processes and four areas [6, 7]. The four processes of SRL are goalsetting, self-monitoring, feedback and control, whilst the four areas an individual can regulate in are cognition, motivation, behaviour and context [6, 7]. The four processes and areas are illustrated in Table 1 *.*

**Table 1:** Areas and processes of self-regulated learning [6, 7]

Self-regulated learning processes	Areas for Self-regulation			
	Cognition	Motivation/affect	Behaviour	Context/environment
Goalsetting/forethought	Setting a criterion to compare progress with	Setting a criterion to compare progress with	Setting a criterion to compare progress with	Setting a criterion to compare with
Self-monitoring	A mechanism used to keep track of their thoughts	A mechanism used to keep track of their motivation	A mechanism used to keep track of their behaviour	A mechanism used to keep track of their environment
Feedback loop	Cyclical processes to monitor the effectiveness of their thoughts	Cyclical processes to monitor their motivational effectiveness	Cyclical processes to monitor the effectiveness of their behaviour	Cyclical processes to monitor the effectiveness of their environment
Control	Selection and adaption of cognitive strategies	Selection and adaption of strategies for managing motivation and affect	Deciding behavioural strategies such as increasing or decreasing effort, persisting or giving up	Selection and adaption of the best contexts for optimal learning

Despite the importance of SRL in medicine, and the significant influence of the environment on student learning [8], SRL cannot be assumed to automatically develop in the clinical learning environment. Medical students will often carry forward learning strategies that worked well in the preclinical years, often to their detriment [9, 10]. Even after graduation, physicians may be quite unskilled at certain aspects of self-regulation, such as global self-assessment [11]. This review will explore what is already known about SRL in medical students during their rotations in the clinical environment.

Although scoping reviews are relatively new [12], they are becoming a more common method to provide an overview or “map” of the literature [13]. This is achieved by investigating the extent of existing research, summarising the findings of all relevant studies and identifying potential gaps in the field [14, 15]. Our scoping review aims to accomplish all three of these reasons.

## Methods

We used the five-stage framework proposed by Arksey and O’Malley in this scoping review which involves (1) identifying the research questions, (2) identifying relevant studies, (3) selecting the relevant studies, (4) charting the data, and (5) collating, summarising and reporting the results. As recommended by the five-stage framework, we did not use a quality appraisal tool for each study, but we followed the explicit process to ensure that our search was replicable, thus increasing the rigour of our findings [14].

### Stage one: identifying the research question

The purpose of our review was to explore the SRL of medical students in the clinical environment. Our research questions were the following:

1. What has been found regarding the self-regulated learning of medical students in the clinical years of their program?
2. How has self-regulated learning in medical students in the clinical learning environment been measured?

### Stage two: identifying relevant studies

A literature search was carried out to identify studies and reports between 1966 and February 2017 using the databases Medline (Ovid), ERIC, EBSCO and Google Scholar. The main search term “self-regulated learning” was combined with key terms and variants of “medical student”, “medical school” and “medical education”. The search identified 911 potential papers. The following criteria were used for the review and selection of the studies: available in English, focused on SRL in the medical clinical learning environment and relevant to the topic after review of abstracts. Articles investigating similar but different theories such as self-directed learning were not included due to conceptual differences [16, 17]. The first author (KC) screened the titles and abstracts for relevance to the research questions, and 890 articles were excluded as they did not meet the inclusion criteria.

### Stage three: study selection

**Fig. 1:**
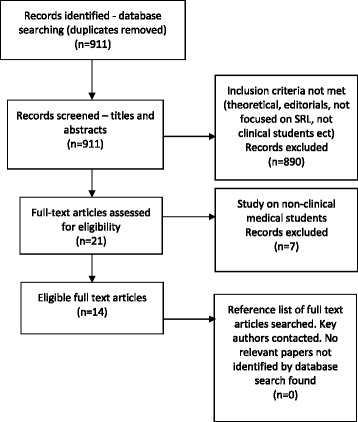
Flowchart of Search Results. Figure 1 Flowchart of Search Results. A flowchart demonstrating this scoping review’s search results

The full texts of the remaining 21 articles were read by KC and 7 articles were excluded as the studies focused solely on non-clinical medical students. The reference lists of all selected publications were then hand searched for any additional relevant studies. Authors of key papers were also contacted for relevant reports or publications. Fourteen articles were selected for inclusion in the review (see Fig. 1).

### Stage four: data charting and collation

The data extraction framework was developed by all authors and implemented by KC. Extracted data from the articles included: authors, date of publication, country of study, study population, study design, instrument used, a brief summary of the primary finding as well as areas and processes of SRL explored in the study. The findings are detailed in Table 2.

**Table 2:** Summary of included studies

Authors	Year	Country	Population and sample	Study design	Instruments used	Primary Findings/ Key findings relevant to the scoping review question/s	Processes of SRL theoretical framework explored (areas of self-regulation explored)
Turan, Demirel and Sayek	2009	Turkey	862 preclinical and clinical medical students from 4 medical schools with different curriculum models	Cross-sectional	Self – Regulated Learning Perception Scale (author’s own)- internal consistency α = 0.76-0.91Metacognitive Awareness Inventory [72]	• Clinical students had higher scores than preclinical students in planning and goalsetting (*p* = 0.001), strategies for learning and assessment (*p* = 0.043) and overall levels of self-regulated learning (*p* = 0.037). • Students with previous exposure to learner centred methods during high school had higher scores in motivation and action to learning (*p* = 0.017). • There were significant differences in all self-regulating scores across different curriculum models	• Planning (cognition, behaviour) • Control (motivation. Behaviour)
Song, Kalet and Plass	2011	USA	58 3rd year medical students (first clinical year)	Cross-sectional	Self–Regulation Measure for Computer–based learning (authors’ own)- Measures Strategies Use (SU) and Strategies Frequency (SF)- high internal consistency (SU α = 0.96-0.99, SF α = 0.97-0.99)- high inter-rater reliability (Interclass correlations of SU = 0.93 (95% CI: 0.89-0.95), Interclass correlations of SF = 0.96 (95% CI: 0.91-0.98)	Strategies Use and Strategies Frequency were significantly correlated with USMLE step 1 scores (*p* < 0.01)	• Planning (cognition, behaviour, environment) • Self-monitoring (cognition, behaviour) • Control (cognition, behaviour, environment)
Sobral	2000	Brazil	103 medical students beginning clinical activities	Cohort	10 – item self-report questionnaire used to measure self-reflection in learning (authors own)	• Significant change in the levels of reflection after the intervention (*p* < 0.001). • 4 items of the Course Valuing Inventory had the strongest relationship with reflection scores (relating and making sense of course contents, *r* = 0.46; Achievement of personal goals, *r* = 0.44; Acquiring a clear and integrated notion of learning processes, *r* = 0.36; Sense of self-esteem related to course experience, *r* = 0.34). • Reflection scores post–intervention were significantly correlated with the following Diagnostic Thinking Inventory scores: flexibility in reasoning (*p* < 0.01) structure of knowledge in memory (*p* < 0.001) and total score (*p* < 0.001) • Reflection scores post–intervention were significantly correlated with grade point average (*p* < 0.01)	• Self-monitoring (cognition)
White	2007	USA	36 medical students – 18 from a PBL medical school and 18 from a traditional medical school	Qualitative	Semi-structured interview	• PBL students effectively transitioned into their clerkship. • Traditional curriculum students had difficulty transitioning from the classroom to the clerkship environment – they felt difficult to find their place and assume responsibility for their learning.	• Planning (cognition) • Self-monitoring (cognition, motivation) • Feedback loop (cognition, behaviour) • Control (cognition, behaviour, motivation)
Cleary and Sandars	2011	USA	Seven 3rd year undergraduate medical students	Qualitative	Self – regulated learning microanalysis	• Students who successfully obtained a blood sample on the first attempt had high levels of strategic thinking in planning, goal setting, self-monitoring and self-evaluation. • Four students indicated that the primary goal was to perform the process of venepuncture correctly. • The 2 students who were needed 3 attempts to obtain a blood sample focused on outcome when planning the procedure and did not monitor their progress.	• Planning (cognition, behaviour, motivation) • Self-monitoring (cognition, behaviour, motivation) • Feedback loop (cognition, behaviour, motivation)
Nguyen, Laohasiriwong, Saengsuwan, Thinkhamrop, Wright	2015	Vietnam	623 medical students across 5 academic years	Cohort	The Depression Anxiety and Stress Scales 21 items [73] the Motivated Strategies for Learning Questionnaire [32]	After controlling for the effects of depression T1, anxiety, stress and other demographic covariates, there were significant negative associations between depression scores and: intrinsic goal orientation, task value, control of learning beliefs, self-efficacy for learning, rehearsal, elaboration, organisation, critical thinking, metacognitive self-regulation, time and study environment, effort regulation and help seeking (all *p* < 0.05).	• Planning (cognition, behaviour, motivation, environment) • Self-monitoring (cognition, behaviour, motivation, environment) • Feedback (cognition, behaviour, environment) • Control (cognition, behaviour, environment)
Turan and Konan	2012	Turkey	309 medical students during their surgical clerkship	Cross-sectional	Motivated Strategies for Learning Questionnaire (MSLQ) [32] Case based examinationObjective Structured Clinical ExaminationTutor evaluations	• Significant but weak correlation between overall MSLQ and OSCE scores (*R* = 0.32, R^2^ = 0.10; *p* < 0.018) • Two most important subdimensions of MSLQ for OSCE scores were self-efficacy (*r* = 0.16) and control over learning beliefs (*r* = −0.17) • No correlation between MSLQ and case-based examination • Significant but weak correlation between MSLQ and tutor evaluation scores (*R* = 0.31;R2 = 0.05; *p* < 0.03)	• Planning (cognition, behaviour, motivation, environment) • Self-monitoring (cognition, behaviour, motivation, environment) • Feedback (cognition, behaviour, environment) • Control (cognition, behaviour, environment
Artino, Dong, DeZee, Gilliland, Waechter, Cruess, Durning	2012	USA	304 medical students at different stages of training	Cross-sectional	30 item survey which included:the authors’ own questions3 subscales adapted from the Patterns of Adaptive Learning Scale [74] The metacognition subscale from the MSLQ [32] The procrastination subscale [75] The avoidance–of–helping-seeking subscale [76] Grade point averageClinical pointsExam pointsRemediation referral	• Mastery goal structures were positively correlated with metacognition (*r* = 0.26, *p* < 0.01) • Metacognition was negatively correlated with procrastination (*r* = −0.12, *p* < 0.05)	• Planning (cognition, behaviour, motivation, environment) • Self-monitoring (cognition, behaviour, motivation)
Woods	2011	Canada	313 medical students in their 3rd and 4th year of training (clerkship)	Qualitative	Series of focus groups	• As students felt a lack of learning opportunities during clerkship rotation, they had to choose and create learning opportunities wisely • There was a lack of critical self-reflection in students self-regulated learning activities	• Planning (cognition, behaviour, environment) • Self-monitoring (cognition, behaviour, environment) • Control (environment)
Alegria, Boscardin, Poncelet, Mayfield and Wamsley	2014	USA	15 students on their Longitudinal Integrated Clerkships	Qualitative	Two focus groups	• Students used tablet computers to read content, collect learning resources and access question banks to assess and track their learning. • Students found tablet computers particularly useful for its ability to access learning issues quickly. • Most students did not use tablet computers to access information during face-to-face interactions with patients	• Feedback (cognition) • Control (environment)
Berkhout, Helmich, Teunissen, van den Berg, van der Vleuten and Jaarsma	2015	Netherlands	17 medical clerkship students – 8 from a PBL medical school, 9 from a traditional medical school	Constructivist grounded theory	Semi structured interviews – Day Reconstruction Method + follow up questions	SRL was supported or inhibited by: • Personal factors: emotional control, metacognition, ability to focus, ability to deal with pressure beliefs about learning • Contextual factors: curriculum, patient-related factors, engagement with team, available time • Social factors: familiarity with staff and colleagues, level of guidance/mentorship from these people • Goals: Supervisors setting goals for student could inhibit student SRL. Goals created by the clerkship students supported SRL. • Opportunities: In order to self-regulate, students felt they needed the opportunities to do so • Experienced autonomy: The more autonomy students felt they had, the more they were able to self-regulate • Anticipated outcomes: Students self-regulated their learning when they expected positive outcomes.	• Planning (cognition, behaviour, environment), • Self-monitoring (cognition, motivation, behaviour), • Control (cognition, behaviour, environment)
Lyons-Warren, Kirby and Larsen	2016	USA	56 medical students on their surgical clerkship	Mixed-methods	18 question electronic survey	• Learning goals were important to maximize learning on surgery clerkship. • 73% of students had defined learning goals at the commencement of their rotation. • Low interest among attending residents in the student’s learning goals. • 48% of students felt the faculty was responsible for initiating the conversation to share learning goals, 16% reported the student was responsible and 12% reported that either the faculty or the student could initiate the conversation • Students felt little flexibility in changing the context of their learning. • Students felt varying degrees on discomfort when asking for opportunities to practice procedural tasks and presenting patient histories/examinations	• Planning (cognition) • Control (behaviour, environment)
Berkhout, Hemlich, Teunissen, Van der Vletuen and Jaarsma	2016	Netherlands	14 medical clerkship students in their 4th, 5th and 6th year	Grounded theory	Interview	Compared to novice learners, experienced learners were: • More proactive in their learning • Less dependent on their peers for support, • More dependent on consultants to help them have adequate learning opportunities • More likely to communicate personal goals with seniors • Less affected by residents in their SRL • More affected by nurses in their SRL However, not all senior students reported learning like an experienced learner.	• Planning (cognition, behaviour, environment) • Feedback (cognition, behaviour, environment) • Control (cognition, behaviour, environment)
Berkhout, Teunissen, Helmich, Exel, Vleuten, Jaarsma	2017	Netherlands	74 clerkship medical students in their 4th, 5th or 6th year.	Q methodology	52 Q-sort statements (author’s own instrument)	Five patterns were retained: • Engaged: actively shape their learning and are motivated to learn from every situation and in a SRL fashion • Critically opportunistic: learn mainly through social interaction, but otherwise are not effortful in their learning. • Uncertain: overwhelmed, frightened, passive and a reactive behavioural pattern. • Restrained: want to learn, but hesitant to include others due to fear of appearing inferior. • Effortful: want to work hard, but depend on others to guide them as they are not capable to structure their learning environment.	• Planning (cognition, behaviour, environment) • Self-monitoring (cognition, behaviour, environment) • Feedback (cognition, behaviour) • Control (cognition, motivation, behaviour, environment)

### Stage five: summarising and reporting the results

The following Results section summarises and discursively reports the review findings.

## Results

### What have studies found regarding self-regulated learning in the medical clinical years?

There were 3 major findings regarding SRL in the clinical years: 1) changes in SRL occur in the clinical learning environment, 2) higher levels of SRL are associated with higher academic achievement, more success in clinical skills and better mental health outcomes and 3) certain factors can influence SRL levels. These are further explained below.

### Changes in self-regulated learning occur in the clinical learning environment

Three studies qualitatively explored how students adapt to different clinical environments. White found that students from a PBL curriculum adapted more effectively and used more SRL skills when transitioning to the clinical environment than students from a traditional curriculum [18]. In Woods et al.’s study, students showed high levels of SRL as they adapted to a surgical rotation but lacked critical self-reflection [10]. Berkhout et al.’s observed in their study that experienced students required less support and were more likely to create their own learning goals compared to novice students [19].

### Self–regulated learning and academic achievement

Five studies reported correlations between levels of SRL and academic achievement during clerkship [20–24]. Sobral’s and Song et al.’s studies found associations between aspects of SRL and achievement in written exams during the clinical years [21, 22], whilst Turan and Konan found no such relationship between SRL scores and written examination results [20]. In regard to success in clinical skills three studies found associations [20, 22, 23]. Turan and Konan found weak correlations between SRL scores and Objective Structured Clinical Examination (OSCE) results [20], Sobral found associations between SRL scores and diagnostic skill [22] and Cleary et al. found a link between SRL and success in cannulation [23]. One study found SRL was negatively correlated with procrastination and positively correlated with mastery goal structures, two variables linked with academic success [24].

### Self-regulated learning and mental health

Nguyen et al. [25] observed that most SRL scores were negatively associated with depression.

### Factors influencing self-regulated learning levels

This review found the SRL of medical students can be affected by a wide range of variables [5, 18, 22, 26–28]. Two studies found that experience was linked with higher levels of SRL [19, 26]. Alegria et al.’s study suggests that technological resources such as tablet computers can further cultivate SRL levels by allowing students to access clinical and educational information [5]. Another study reports that a PBL curriculum can support the SRL of students especially during the transition to the clinical environment [18, 19]. Sobral’s study suggests reflection, one aspect of SRL, can be systematically improved through specific interventions [22]. Berkhout et al.’s 2015 study reports the SRL of clinical medical students is influenced by a range of variables including personal, contextual, social factors as well as experienced autonomy [27]. Lyons-Warren et al., suggest SRL is hindered when students feel there is a lack of flexibility when pursuing their learning needs [28]. Berkhout et al.’s 2017 study proposes five SRL patterns exist for clinical students, with each pattern requiring a unique approach to support learning [29].

### How has self-regulated learning in medical students in the clinical learning environment been measured?

Methods used to study SRL were varied and included qualitative, quantitative and mixed-method approaches.

Qualitative processes such as semi structured interviews and focus groups were used in five studies [5, 10, 18, 19, 27]. One other study used microanalytic assessment [23]. Microanalysis was originally used to explore the differences in SRL between novice and professional sportsmen [30], and is a structured process which involves verbal responses to open–ended questions, targeting forethought, performance and reflection processes during a specific task. The responses are recorded verbatim then coded [31].

Quantitative approaches included the Self-Regulated Learning Measure for Computer-based learning (SRMC) [21], the Self–Regulated Learning Perception Scale [26] and the Motivated Strategies for Learning Questionnaire (MSLQ) or a modified version of this instrument [20, 25].

The SRMC tests the use and frequency of 10 SRL subcategories (self-evaluation, organizing and transforming, goal setting and planning, seeking information, keeping records and monitoring, environmental structuring, self-consequences, rehearsing and memorizing, seeking social assistance, and reviewing records) and two classes of non-self regulation behavior (will power and non-applicable statements). The tool has high internal consistency and high inter-rater reliability [21].

The Self–Regulated Learning Perception Scale uses 41 items to measure 4 domains; 1) motivation and action to learning 2) planning and goalsetting 3) strategies for learning and assessment and 4) lack of self–directedness [26]. The scale is highly reliable.

Three articles used the MSLQ [20, 24, 25], which was developed by Pintrich in 1991 [32]. The MSLQ contains 81, 7-point Likert type scale questions measuring 15 subscales of SRL. The MSLQ has variable internal consistency depending on the subscale and high validity [2].

One study used Q-methodology, a method that uses features of both quantitative and qualitative measures [29]. In the Q-methodology participants sort a set of statements along a continuum of a fixed grid (from not at all applicable to me, to very applicable to me) and are asked to explain their rationale for their sorting choices. Similar patterns are identified in the population, and the resulting patterns are interpreted and described as shared perspectives.

## Discussion

In this review, the literature on the SRL of medical students in the clinical environment was systematically searched and summarized. All but three studies [18, 26, 27] were conducted amongst students from a single institution.

### Coverage of the SRL theoretical framework by the included studies

The four processes and four areas of SRL were well covered by the included studies (see Table 3). Whilst some studies focussed on explicit parts of SRL such as increasing control of the learner’s environment [5], other studies through the use of instruments such as the MSLQ explored SRL through a wider lens [20, 25]. Examining studies outside the SRL literature would be useful to further characterise specific aspects of SRL, for example how clinical medical students have appraised the effectiveness of the clinical learning environment [33, 34].

**Table 3:** Areas and processes of SRL explored in studies

Self-regulated learning processes	Areas for Self-regulation			
	Cognition	Motivation/affect	Behaviour	Context/environment
Goalsetting/forethought	Turan et al. (2009) [26] Song et al. (2011) [21] White (2007) [18] Cleary and Sandars (2011) [23] Turan and Konan (2012) [20] Nguyen et al. (2015) [25] Artino et al. (2012) [24] Woods (2011) [10] Berkhout et al. (2015) [27] Lyons-Warren et al. (2016) [28] Berkhout et al. (2016) [19] Berkhout et al. (2017) [29]	Cleary and Sandars (2011) [23] Nguyen et al. (2015) [25] Artino et al. (2012) [24] Turan and Konan (2012) [20]	Turan et al. (2009) [26] Song et al. (2011) [21] Cleary and Sandars (2011) [23] Nguyen et al. (2015) [25] Turan and Konan (2012) [20] Artino et al. (2012) Woods (2011) [10] Berkhout et al. (2015) [27] Berkhout et al. (2016) [19] Berkhout et al. (2017) [29]	Song et al. (2011) [21] Nguyen et al. (2015) [25] Turan and Konan (2012) [20] Artino et al. (2012) [24] Woods (2011) [10] Berkhout et al. (2015) [27] Berkhout et al. (2016) [19] Berkhout et al. (2017) [29]
Self-monitoring	Turan et al. (2009) [26] Song et al. (2011) [21] Sobral (2000) [22] White (2007) [18] Cleary and Sandars (2011) [23] Nguyen et al. (2015) [25] Turan and Konan (2012) [20] Woods (2011) [10] Artino et al. (2012) [24] Berkhout et al. (2015) [27] Berkhout et al. (2017) [29]	White (2007) [18] Cleary and Sandars (2011) [23] Nguyen et al. (2015) [25] Turan and Konan (2012) [20] Artino et al. (2012) [24] Berkhout et al. (2015) [27]	Turan et al. (2009) [26] Song et al. (2011) [21] Cleary and Sandars (2011) [23] Nguyen et al. (2015) [25] Turan and Konan (2012) [20] Artino et al. (2012) [24] Woods (2011) [10] Berkhout et al. (2015) [27] Berkhout et al. (2017) [29]	Nguyen et al. (2015) [25] Turan and Konan (2012) [20] Woods (2011) [10] Berkhout et al. (2017) [29]
Feedback loop	White (2007) [18] Cleary and Sandars (2011) [23] Turan and Konan (2012) [20] Alegria et al. (2014) [5] Berkhout et al. (2016) [19] Berkhout et al. (2017) [29]	Cleary and Sandars (2011) [23] Nguyen et al. (2015) [25]	White (2007) [18] Cleary and Sandars (2011) [23] Nguyen et al. (2015) [25] Turan and Konan (2012) [20] Berkhout et al. (2016) [19] Berkhout et al. (2017) [29]	Nguyen et al. (2015) [25] Turan and Konan (2012) [20] Berkhout et al. (2016) [19]
Control	Song et al. (2011) [21] White (2007) [18] Nguyen et al. (2015) [25] Turan and Konan (2012) [20] Berkhout et al. (2015) [27] Berkhout et al. (2016) [19] Berkhout et al. (2017) [29]	White (2007) [18] Berkhout et al. (2017) [29]	Turan et al. (2009) [26] Song et al. (2011) [21] White (2007) [18] Nguyen et al. (2015) [25] Turan and Konan (2012) [20] Berkhout et al. (2015) [27] Lyons-Warren et al. (2016) [28] Berkhout et al. (2016) [19] Berkhout et al. (2017) [29]	Song et al. (2011) [21] Nguyen et al. (2015) [25] Turan and Konan (2012) [20] Woods (2011) [10] Alegria et al. (2014) [5] Berkhout et al. (2015) [27] Lyons-Warren et al. (2016) [28] Berkhout et al. (2016) [19] Berkhout et al. (2017) [29]

### Changes in self-regulated learning occur in the clinical learning environment

Two studies that investigated clinical transition highlighted the importance of self-regulating learning to maximise experiential learning [10, 18]. The importance of self–regulated learning during the transition to clerkship is not surprising as supervisors are often pre-occupied with patient care and sometimes not interested to teach [35, 36]. However, this scoping review did not find any quantitative studies that explored the preclinical to clinical transition using a validated tool. Thus future studies could explore this transition using a validated quantitative approach to provide further insights into the topic.

Beyond the transition period, Berkhout et al.’s study suggests SRL continues to develop in the clinical environment, with novice and experienced learners having different needs to support their SRL. The study notes that novice students require more support from others, specifically residents and peers, to help them formulate learning goals and navigate the new learning environment. In the broader medical education literature, the significance of resident teaching is mixed, with some studies highlighting the correlation between resident teaching and medical student learning [37] and academic performance [38], whilst other studies not finding such an effect [39, 40].

### Self–regulated learning, academic achievement and mental health

This review indicates that higher levels of SRL may be beneficial for medical students due to its positive correlation with academic achievement and clinical skills [20–24] as well as its negative association with depression [25].

The positive correlation between SRL and diagnostic and clinical skills is important for students not just when they are in medical school but also as practising physicians. Furthermore, as doctors must maintain their competencies and skills throughout their careers in order to consistently meet the high standards of patient care [41–43], they must develop an awareness of their learning needs and use appropriate learning strategies to achieve their goals. As lifelong learners, doctors should adopt SRL and be motivationally, behaviourally and meta-cognitively proactive in their own learning process [44–46]. Developing SRL in medical students in the clinical environment is important not only for the short term, so that medical students may achieve better grades and clinical proficiency, but also for the long-term so that graduates can participate in lifelong learning and provide patients with effective care.

The negative association between SRL and depression is significant because medical students as a whole face higher levels of psychological stress than the general population [47, 48], with studies suggesting that rates of depression increase during the clinical years [49, 50]. Moreover some evidence suggests that mental distress during medical school is associated with problems as physicians [51, 52], which might negatively affect patient care [53]. Although the link between SRL and decreased depression is correlational and not causative, the association between aspects of SRL and mental health has been found in other studies involving preclinical medical students [54] as well as in studies outside medical education [55, 56]. Thus it is may be relevant to promote SRL strategies to help students optimise their mental well-being.

### What factors influence the self-regulated learning levels of medical students in the clinical learning environment?

In their study, Turan et al. found that medical students in the clinical years appeared to intrinsically have higher levels of SRL than preclinical medical students [26]. Although it is reassuring to believe self-regulating learning naturally develops and increases as students progress through medical school and postgraduate training, caution must applied when interpreting the results as the study explored the changes in SRL in separate cohorts rather than following its development in the same student cohort. Indeed numerous authors in the broader literature have suggested that self-regulated learners are not always successful when left to develop their own strategies [57–60].

Finally it appears that at least some aspects of SRL can be targeted and improved, with one study suggesting that levels of reflection in students can be increased after an intervention. This finding that SRL can be improved by certain interventions is supported by research outside the transitions literature [42, 61–63], with one author suggesting that explicitly teaching metacognition is efficacious [61].

### How has self-regulated learning in medical students in the clinical learning environment been measured?

Several approaches have been used to measure SRL in clinical medical students and there appears to be no single best approach with each approach having its advantages and disadvantages. For example, studies using the qualitative approach were effective in identifying and exploring contextual factors related to SRL in the clinical environment whereas the quantitative approach could identify the strengths of associations to test hypotheses. Two of the three studies using quantitative methods developed their own instruments specifically for use in the clinical context [21, 26]. In terms of microanalytic assessment, the tool has been reported in the literature to be effective in examining motor and cognitive skills that have a clear beginning, middle and end section [64]. Thus microanalytic assessment appears to be a suitable method to analyse the SRL of students during specific tasks such as taking a history and examining a patient or skills such as cannulation, but not to measure global SRL that may occur with informal learning in the clinical environment. Q methodology measures subjective experiences through features of qualitative and quantitative approaches, and has been reported to be a more robust technique than Likert-type scales to study attitudes in health education [65].

Studies which investigated SRL across multiple cohorts reported the cross-sectional design as a limitation. These authors suggest longitudinal studies may allow the transition to be better measured. Studies using questionnaires as their collection tool reported reliability and validity limitations, especially as they measured self-reported learning behaviours, which are subject to social desirability and recall bias. However each survey instrument had reasonable psychometric properties. Some studies also reported the possibility of selection bias either due to small sample sizes [24] or due to inherent differences in the selected students [18]. Future studies could thus consider using longitudinal study design using a range of methods to better understand how SRL develops and is maintained in the clinical environment.

Not identified in our search were novel qualitative methods used in the broader SRL literature, such as think-aloud protocols [66, 67], video-taped events [68], structured study-diaries [69] and computer trace analysis [70, 71]. Future studies could use these novel methods to measure the SRL of clinical medical students.

### Limitations

This review was restricted to articles published in the English language and those concerning the medical profession. We also did not include studies with similar terms to SRL such as self-directed learning in our review due to conceptual differences in perspectives and constructs. We did include medical and educational databases as well as Google Scholar to widen the search to where SRL research might be catalogued. Future research should consider including nursing and other allied health professionals as learning in the clinical environment is also a core part of health professional education and findings about effective learning could be transferrable across professions. As this article is a scoping review and not a systematic review, we did not critically appraise the selected studies. We did however locate findings which addressed our aims of examining the nature and extent of the literature, and to identify potential future research directions.

## Conclusion

We explored what is known about medical students in the clinical environment, specifically in relation with SRL. We found several relevant papers, with most published in the last 5 years, suggesting a growing interest in SRL in the clinical environment in medical education. Although most had a cross-sectional or qualitative design, quantitative approaches may yield complementary insights, with longitudinal research being needed to examine how SRL develops and is maintained in clinical learning environments. Additionally future studies could consider using novel qualitative methods to explore the SRL of medical students in the clinical environment.

## Abbreviations

MSLQ: Motivated Strategies for Learning Questionnaire
OSCE: Objective Structured Clinical Examination
PBL: Problem-Based Learning
SRL: Self-regulated learning
SRMC: Self-regulated Learning Measure for Computer-based Learning
